# Transcatheter aortic valve replacement in a quadricuspid aortic valve: a systematic review of case reports

**DOI:** 10.3389/fcvm.2025.1572251

**Published:** 2025-05-30

**Authors:** Mostafa A. Khalifa, Hashim Talib Hashim, Aya Ahmed Shimal, Fathima Raahima Riyas Mohamed, Srinithi Ragunathan, Ahmed Sermed Al Sakini, Mohamed H. Elbadawi, Mohammed Rushdhi Irfan, Ibrahim Moqbel, Marwah Mohammed Almualed, Mohammedbaqer Al-Ghuraibawi, Batool S. Al-Aboudi

**Affiliations:** 1 Faculty of Medicine, Cairo University, Cairo, Egypt; 2 College of Medicine, University of Warith Al-Anbiyaa, Karbala, Iraq; 3 College of Medicine, University of Baghdad, Baghdad, Iraq; 4 College of Medicine, Alfaisal University, Riyadh, Saudi Arabia; 5 Panimalar Medical College and Research Institute, Chennai, India; 6 Faculty of Medicine, University of Khartoum, Khartoum, Sudan; 7 College of Medicine, King Abdulaziz University, Jeddah, Saudi Arabia; 8 Faculty of Medicine, University of Kufa, Najaf, Iraq

**Keywords:** transcatheter aortic valve replacement, quadricuspid aortic valves, aortic stenosis, procedural success, QVA

## Abstract

**Background:**

While Transcatheter Aortic Valve Replacement (TAVR) is now a standard treatment for severe aortic stenosis, its use in patients with quadricuspid aortic valves (QAV) presents unique challenges. This review analyzes current evidence to guide clinicians in managing aortic stenosis in this complex valve morphology.

**Method:**

Following PRISMA guidelines, a comprehensive literature search was conducted across multiple databases up to August 15, 2024. A descriptive pooled analysis of individual participant data using a one-stage approach was performed, along with appropriate univariate tests, focusing on 30-day mortality and procedural success, with secondary outcomes including paravalvular leak incidence, pacemaker insertion, hemodynamic changes, and NYHA functional class improvement.

**Results:**

A total of 11 case reports/series were analyzed, involving 17 adult patients with QAV. Participants had a mean age of 73.80 ± 5.07 years. The mean left ventricular ejection fraction was 41.6%, and the mean annulus area was 595.5 mm^2^. Most patients (64.7%) underwent transfemoral procedures, with nearly 70% receiving a J-valve or Edwards SAPIEN 3 device. All procedures were largely successful, though 29.4% experienced leakage or regurgitation. Aortic pre-dilation was done in 41.2% of cases. The mean procedural duration was 102 min, with a fluoroscopic duration of 15 min. No patients experienced aortic post-dilation, and one (5.8%) had an atrioventricular block within 30 days post-procedure.

**Conclusion:**

TAVR is an effective and growing treatment for high-risk patients with aortic valve disease, including those with QAV. While it has high success rates and challenges (i.e., post-operatively). Future studies should focus on long-term valve durability.

## Introduction

Transcatheter aortic valve replacement (TAVR) has revolutionized the management of severe aortic stenosis, particularly in patients at high or intermediate surgical risk (1). Traditional surgical aortic valve replacement (SAVR) has long been the standard treatment for symptomatic severe aortic stenosis. However, the advent of TAVR has provided a less invasive alternative with promising outcomes. This is particularly significant given the aging global population and the growing prevalence of aortic stenosis. TAVR offers a viable option for those who are not ideal candidates for open-heart surgery due to comorbid conditions or frailty (1, 2).

Over time, the indications for TAVR have expanded to include intermediate-risk patients and, more recently, those at low surgical risk (3). This expansion has been supported by a series of clinical trials demonstrating the safety and efficacy of TAVR across a range of patient populations. The key studies, such as PARTNER (Placement of Aortic Transcatheter Valves) (1) and CoreValve (2) studies, have established TAVR as a transformative approach to treating aortic stenosis.

Quadricuspid aortic valve (QAV) is a rare congenital anomaly where the aortic valve has four cusps instead of the usual three. This condition is estimated to occur in less than 1% of the general population (4). QAV can be associated with various clinical issues, including aortic stenosis, regurgitation, or a combination of both (5). The presence of four cusps rather than three introduces unique anatomical challenges that can complicate both the diagnosis and treatment of aortic valve disease. The rarity of QAV contributes to a limited understanding of its implications for TAVR. The four cusps can lead to variations in valve anatomy, which may affect the choice of prosthetic valve, its positioning, and the risk of complications. Furthermore, QAV can be associated with other congenital heart defects, which may further complicate the management of the condition (4, 6).

The literature on TAVR in QAV patients is sparse and often limited to small case series or single-center studies. While some reports suggest that TAVR can be successfully performed in patients with QAV, there is a lack of comprehensive, large-scale data to guide clinical practice. Existing studies have highlighted both successful outcomes and complications specific to this valve morphology, such as issues related to valve sizing, deployment, and the potential for paravalvular leak. Despite these individual studies, a systematic and comprehensive review of TAVR outcomes in QAV patients is lacking.

This systematic review and meta-analysis aim to fill this gap by synthesizing the available evidence on the outcomes of TAVR in patients with QAV. The objectives are to evaluate the procedural success rates, identify common complications, and assess long-term outcomes associated with TAVR in this unique patient population. By aggregating data from multiple studies, this review seeks to provide a clearer understanding of the efficacy and safety of TAVR for patients with QAV.

## Methods

**Figure 1:**
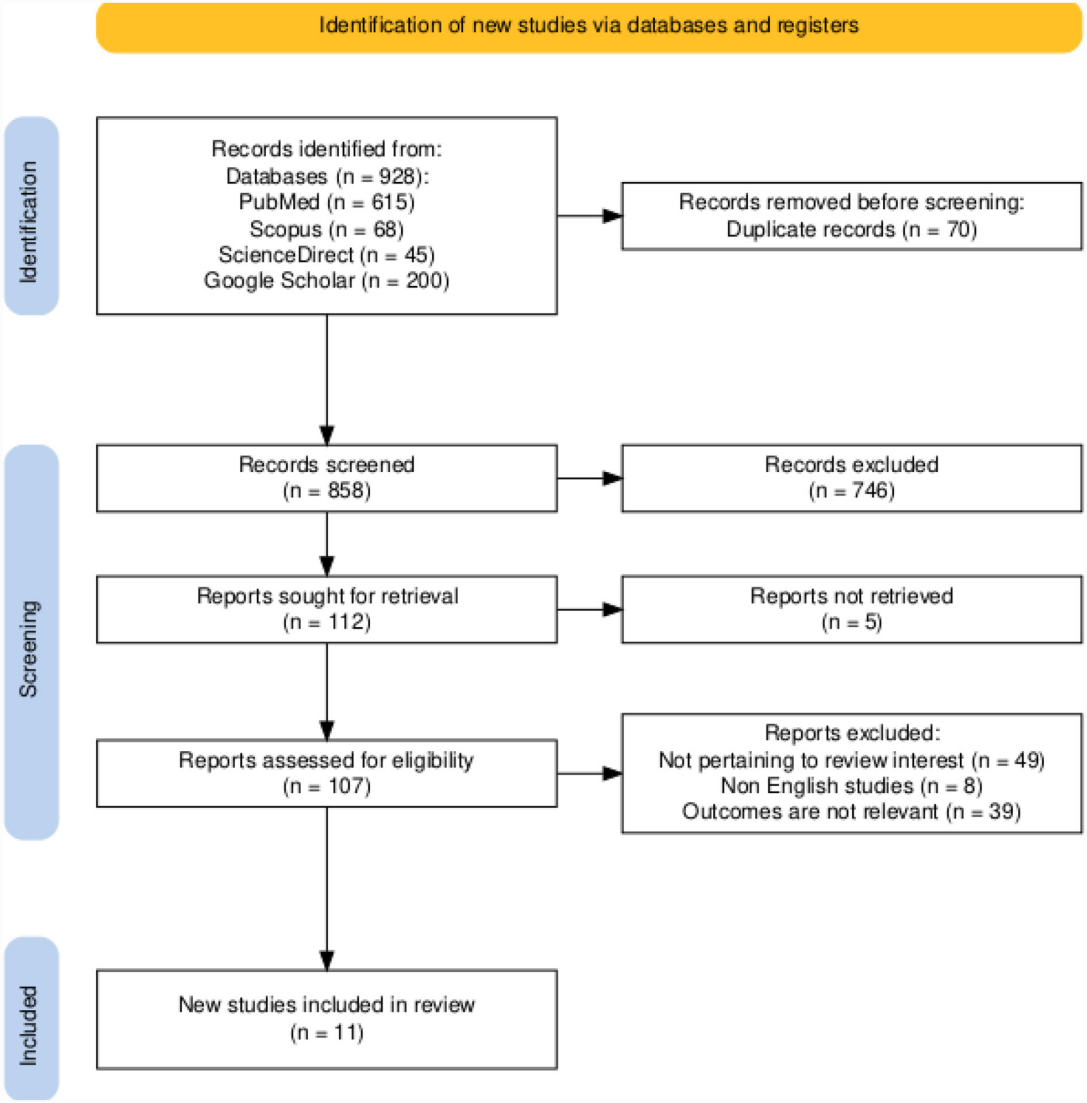
PRISMA flowchart of the present systematic review and meta-analysis.

This review was undertaken and reported in accordance with the preferred reporting items for systematic review and meta-analyses (PRISMA) guidelines (7) (Figure 1).

### Search strategies and information sources

A comprehensive literature search was conducted across multiple electronic databases, including (PubMed, Scopus, ScienceDirect and Google scholar) up to August 15, 2024. A combination search of subject terms was applied. Subject terms included: (“quadricuspid aortic valve” OR “QAV” OR “aortic valve abnormality” OR “congenital aortic valve disease” OR “congenital heart disease”) AND(“transcatheter aortic valve replacement” OR “TAVR” OR “TAVI” OR “aortic valve intervention”)AND (“procedural success” OR “mortality” OR “morbidity” OR “hemodynamic improvement” OR “complications” OR “quality of life”).

### Study selection and eligibility criteria

A two-stage screening process was used to select eligible studies. First, two reviewers screened titles and abstracts independently (1 and 2) to identify potentially relevant studies based on pre-defined inclusion criteria. Disagreements were resolved through consensus or consultation with a third reviewer (3). Second, the same two reviewers independently assessed full-text articles of potentially eligible studies using the pre-defined inclusion and exclusion criteria. Any discrepancies were again resolved through consensus or consultation with the third reviewer.

### Inclusion criteria

- Case reports or retrospective case series.
- Patients diagnosed with QAV.
- Underwent TAVR procedure.
- Reported at least one relevant outcome (e.g., procedural success, mortality, paravalvular leak, hemodynamic parameters, NYHA functional class).

### Exclusion criteria

- Non-English studies.
- Studies involving patients with other significant valvular pathologies besides QAV that could confound the results.
- Studies that did not provide sufficient data for extraction.
- Review articles, editorials, letters, or abstracts without full-text articles.
- Studies using animal models.

### Data extraction

Four reviewers (Reviewer 1 and Reviewer 2,3,4) extracted data separately using a standardized data extraction form. A variety of information was extracted, including study characteristics (author, year, publication type, country, sample size), patient characteristics (age, sex, QAV morphology, surgical risk score), procedural details (type of valve, approach), and outcomes (procedural success, 30-day mortality, paravalvular leak, hemodynamic parameters, NYHA functional class, length of hospital stay, duration of follow-up, complications). Any disagreements were handled by debate, consensus, or consultation with the third reviewer.

### Assessment of methodological quality and risk of bias

The methodological quality of the included studies was evaluated using the JBI quality assessment tool (8). The JBI tool evaluates the quality of case reports across multiple domains: demographics, history timeline, clinical condition, diagnostic test, intervention description, post-intervention, adverse events, take home lessons. Each domain receives a final review categorized as not reported, good, fair, or poor. Ultimately, each study receives a conclusive assessment based on the evaluations reported across all domains. The quality assessment involved two reviewers [XX and XX]. Discrepancies among reviewers were addressed through discussion and the senior author's advice.

### Statistical analysis

Individual participants' data analysis was used. The analysis approach was a one-stage approach that directly utilized the data from the articles without clustering due to small sample size per each article. Data cleaning and organization was performed using SPSS version 26. Normality test was performed for continuous variables which yielded normally distributed data (non-significant result in Shapiro-wilk test –*p*-value > 0.05). Frequency and descriptive statistics were used to present the patients’ characteristics and surgical characteristics. Data was presented as numbers and percentages or mean and standard deviations. Univariate analysis was performed for post-surgical aortic valve status because it was the only outcome variable with a missing percentage less than 50% to ensure proper representation of the data. Variables with missing percentages less than 50% and can form 2 × 2 tables have been introduced in the univariate analysis. The univariate analysis was performed using the Maximum likelihood ratio test for categorical variables, as it was described as the best approach for small sample sizes (Here, Here). The Mann–Whitney test was used for continuous variables, as it was described to be useful for sample sizes less than 20 (Here). In all tests the used level of significance is 0.05.

## Results

The initial database search yielded 11 case reports/series which were included in the analysis. All articles included single patient except: Heberto Aquino-Bruno (2 patients), Yang Liu (9) (5 patients) and Daisuke Sato (2 patients). 3 articles were from China, 4 from Japan, 1 from Mexico, 1 from France, 1 from Australia and 1 from South Korea. All articles addressed adults with quadricuspid aortic valves. Generally, all articles reported good outcomes after Transcatheter Aortic Valve Replacement (TAVR) with some degree of leakage/regurgitation across some patients (Table 1).

**Table 1:** Characteristics of the included studies.

Study ID	Design	Sample size	Site	Population	Interventions	Results and outcome
Yu Han, 2021 (9)	case study	1 patient	China	70-year-old man with symptomatic aortic stenosis and regurgitation	Transcatheter Aortic Valve Replacement (TAVR)	The 26-mm valve resulted in paravalvular leak (PVL), and the implantation of a second valve reduced the PVL from severe to moderate.
Tomoki Fukui, 2020 (10)	case study	1 patient	Japan	74-year-old woman with severe aortic stenosis and moderate regurgitation	Transcatheter Aortic Valve Replacement (TAVR) using a 23 mm Edwards Sapien 3 valve	Successful TAVR, postoperative aortic mean pressure gradient decreased to 19 mmHg with mild perivalvular leakage. No cardiac events during a 3-month follow-up
Chaodi Luo, 2021 (11)	Case study	1 patient	China	A 62-year-old male patient with a congenital quadricuspid aortic valve (QAV) with severe aortic regurgitation and mild aortic stenosis.	Transapical aortic valve implantation (TAVI) using the J-Valve system.	Successful implantation with no complications, significant symptom relief, and normal function of the artificial aortic valve during follow-up. Left ventricular ejection fraction (LVEF) improved from 59% to 64% after 6 months.
Samuel Sidharta, 2015 (12)	case study	1 patient	Australia	A 90-year-old man with worsening exertional dyspnea and a history of severe aortic stenosis and moderate regurgitation	Transcatheter aortic valve implantation (TAVI) using a 27-mm PORTICO transcatheter heart valve	Successful TAVI with mild post-implantation regurgitation. Significant symptom improvement (NYHA Class II) at 1-month follow-up
Rie Aoyama, 2018 (13)	case study	1 patient	Jap hi an	A patient with severe aortic stenosis (AS) and aortic regurgitation (AR), with a quadricuspid aortic valve (QAV)	Transcatheter aortic valve implantation (TAVI) using the Evolut R valve	The mean pressure gradient reduced from 51.5 mmHg to 3.0 mmHg. Diastolic blood pressure increased from 35 to 51 mmHg. There was a trivial paravalvular leak post-procedure.
Cheol woong you, 2014 (14)	Case study	1 patient	South Korea	An 80-year-old male patient with a quadricuspid aortic valve (QAV) stenosis, severe left ventricular systolic dysfunction, and multi-organ failure	Balloon aortovalvuloplasty as a bridge therapy followed by transcatheter aortic valve implantation (TAVI) using a 26 mm Edwards SAPIEN XT valve.	Successful recovery from multi-organ failure and proper positioning of the implanted valve confirmed by computed tomographic angiography (CTA). The patient recovered
Heberto Aquino-Bruno,2024 (15)	case report	2 patients	Mexico	Patient 1: 2 old men. One with 81 years old and one with 79 years old. Both with severe aortic stenosis and aortic insufficiency.	Transcatheter Aortic Valve Replacement (TAVR) using a 23-mm Edwards SAPIEN 3 valve	Successful TAVI with mean gradient reduced and reduced velocity. No complications were noted and no conduction alteration.
Yang Liu 2022 (9)	case report	5 patients	China	Five patients with a mean age of 73.8 years (range 69–82 years); four patients were male. They had severe aortic regurgitation for four patients and severe aortic stenosis and moderate regurgitation in one patient.	Transapical aortic valve implantation (TAVI) using the J-Valve system (27 mm or 29 mm) in four patients and the other patient with AS received a 32 mm Venus-A prosthesis	Procedural success was achieved in all patients with heart failure symptoms improved The patient with AS was detected by a trivial paravalvular leak, and his mean pressure gradient decreased from 50.5 to 6.0 mmHg in the patient with AS
Henri Benkemoun,2020 (16)	case study	1 patient	France	An 87-year-old woman with severe aortic stenosis, and history of unstable angina related to coronary disease	Transcatheter Aortic Valve implantation (TAVR) using a 23-mm Edwards Sapien 3 valve	The immediate result of the TAVI was as good as possible, with no aortic regurgitation and no coronary damage
Masao Takahashi, 2022 (17)	case report	1 patient	Japan	An 84-year-old woman with severe aortic stenosis and regurgitation	Transcatheter Aortic Valve Replacement (TAVR) using a 23 mm SAPIEN 3 valve	After TAVR, the patient became hemodynamically unstable due to 99% stenosis of the left main coronary artery, which was successfully treated with stenting
Daisuke Sato, 2022 (18)	case study	2 patients	Japan	Two women, aged 83 and 75, both with quadricuspid aortic valve stenosis	Transcatheter Aortic Valve Implantation (TAVI) using a 23-mm SAPIEN 3 valve	Successful TAVI with good postoperative results (mean gradients of 8.0 and 8.6 mmHg) with paravalvular regurgitation in one patient.

### Patients' characteristics

Of 17 quadricuspid aortic valve (QVA) participants, the mean age was 73.80 ± 5.07. The mean weight was 63.2 ± 9.09. Most of the patients were from China (7, 41.2%) and Japan (5, 29.4%). More than half of the patients were males (11, 64.7%). More than half of the patients (11, 64.7%) had both aortic stenosis and regurgitation with type B (Hurwitz and Roberts classification) being the most common reported QVA type (5, 29.4%). Almost 70% of the patients (12, 70.6%) presented with either reported heart failure symptoms or progressive dyspnea. The most common reported comorbidities were: coronary artery disease (5, 29.4%) and hypertension (5, 29.4%). More than half of the patients (7, 58.3%) had calcifications in the aorta. Among the patients with reported information: mean of left ventricular ejection fraction was 41.6 ± 7.2% and the mean of Annulus area was 595.5 ± 69.8 mm^2^ (Table 2).

**Table 2:** Baseline characteristics of the participants (*N* = 17).

Variable		Frequency	Percentage
Age (years)		73.80 (mean)	5.07 (SD)^a^
Body weight (kg)		63.2 (Mean)	9.09 (SD)^a^
Location	Japan	5	29.4%
	Mexico	2	11.8%
	South Korea	1	5.9%
	China	7	41.2%
	France	1	5.9%
	Australia	1	5.9%
Gender	Male	11	64.7%
	Female	6	35.3%
Functional status of aortic valve	Aortic regurgitation	4	23.5%
	Aortic Stenosis	2	11.8%
	Both aortic stenosis and regurgitation	11	64.7%
QVA type (Hurwitz and Roberts classification)	Not identified	5	29.4%
	A	4	23.5%
	B	5	29.4%
	C	1	5.9%
	D	1	5.9%
	F	1	5.9%
Clinical presentation	Symptoms of heart failure	7	41.2%
	Progressive dyspnea	5	29.4%
	Syncope	1	5.9%
	Symptomatic aortic stenosis and regurgitation	4	23.5%
Comorbidities^b^	Not mentioned	4	23.5%
	Chronic lymphocytic leukemia	2	11.8%
	Chronic obstructive airway disease or asthma	3	17.6%
	Coronary artery disease	5	29.4%
	Pulmonary fibrosis	1	5.9%
	Dyslipidemia,	1	5.9%
	diabetes mellitus	1	5.9%
	Reduced left ventricular function	1	5.9%
	Heart failure	1	5.9%
	Hypertension	5	29.4%
	Recent gastrointestinal bleeding	1	5.9%
	Aortic dissection	1	5.9%
	Cerebral infarction	1	5.9%
Presence of hypertension as co-morbidity	Yes	6	35.3%
	No	11	64.7%
New York Heart Association (NYHA) Functional Classification	class III	7	41.2%
	class IV	3	17.6%
	Missing	7	41.2%
Presence of calcification	Yes	7	58.3%
	No	5	41.7%
Left ventricular ejection fracture (%)	Identified	41.6 (Mean)	7.2 (SD)^a^
	Missing	9	52.9%
The American Society of Thoracic Surgeons (STS) risk score (%)	Identified	7.5 (Mean)	6.3 (SD)^a^
	Missing	10	58.80%
Annulus perimeter (mm)	Identified	87.1 (Mean)	5.1 (SD)^a^
	Missing	8	47.10%
Annulus area (mm^2^)	Identified	595.5 (Mean)	69.8 (SD)^a^
	Missing	7	41.20%
STJ diameter (mm)	Identified	29.7 (Mean)	2.2 (SD)^a^
	Missing	8	47.10%
LCA height (mm)	Identified	10.7 (Mean)	2.9 (SD)^a^
	Missing	7	41.20%
RCA height (mm)	Identified	13.9 (Mean)	4.7 (SD)^a^
	Missing	7	41.20%

a SD, Standard deviation.

b Selection of more than one choice was permitted (reason for the percentages not adding up to 100%).

### Surgical and post-operative characteristics of the participants

More than half of the patients were subjected to a transfemoral approach for the surgery (11, 64.7%). Almost 70% of the patients (12) were subjected to J-valve system device or Edwards SAPIEN 3 device. All procedures resulted generally in a successful outcome based on the surgeon's judgment, with 5 patients suffering from leak/regurgitation (29.4%). 7 patients (41.2%) underwent aortic pre-dilation via Balloon aortovalvuloplasty (3, 17.6%) or balloon dilation (4, 23.5%). The mean procedural duration was 102.0 ± 25.9 min, while the mean fluoroscopic duration was 15.0 ± 3.4 min. Among the reported post-dilation data, all of them (8, 47.1%) didn't have any aortic post-dilation. One patient (5.8%) reported having Atrioventricular block during 30-days follow-up (Table 3).

**Table 3:** Surgical and post-operative characteristics of the participants (*N* = 17).

Variables		Frequency	Percentage
Surgery approach	Transfemoral	11	64.7%
	Transcatheter (TAVR)	1	5.9%
	Transapical	5	29.4%
Total Follow up duration	1 month	1	8.3%
	3 months	1	8.3%
	6 months	4	33.3%
	12–56 months (the median 18)	5	41.7%
	48 months	1	8.3%
Transcatheter aortic valve replacement (TAVR) devices used	J -valve	5	29.4%
	Venus A-valve	2	11.8%
	Edwards SAPIEN 3	7	41.2%
	PORTICO transcatheter heart valve	1	5.9%
	Evolut R valve	1	5.9%
	Edwards SAPIEN XT valve	1	5.9%
Procedural duration (min)	Identified	102 (mean)	25.9 (SD)^a^
	Missing	12	70.6%
Fluoroscopic duration (min)	Identified	15.0 (mean)	3.4 (SD)^a^
	Missing	13	76.5%
Pre dilatation performed	Missing	6	35.3%
	Performed via Balloon aortovalvuloplasty	3	17.6%
	Performed via ballon dilation	4	23.5%
	Not performed	4	23.5%
post dilatation performed	Yes	0	0.0%
	No	8	47.1%
	Missing	9	52.9%
Procedural subjective success	Yes	17	100.0%
	No	0	0.0%
Postprocedural aortic status	Mild paravalvular regurgitation	1	5.8%
	Mild perivalvular leakage	1	5.8%
	Moderate paravalvular leakage	1	5.8%
	Normal	12	70.6%
	Trivial paravalvular leakage	1	5.8%
	Trivial to mild regurgitation	1	5.8%
Length of hospital stay (days)	Identified	6 (Mean)	2 (SD)^a^
	Missing	10	58.8%
In-hospital and 30-day complications	A–V block	1	5.8%
	Coronary artery stenosis during TAVR	1	5.8%
	Missing	11	64.7%
	No complications	4	23.5%
1-year Left ventricular ejection fracture (%)	Identified	49.8 (Mean)	3.4 (SD)
	Missing	12	70.6%
1-year New York Heart functional class (NYHA)	Class 1	2	11.8%
	Class 2	2	11.8%
	Class 3	1	5.9%
	Missing	12	70.60%

a SD, standard deviation.

### Factors affecting postprocedural aortic valve status

The patients' baseline and surgical characteristics were plotted against post-surgical aortic valve status. Age, gender, Functional status of the aortic valve and QVA classification weren't found to be significant with the valve status. Moreover, the devices used weren't found to affect the aortic valve status significantly. However, the used surgical approach was found to be a significant contributor to the status, with the transcatheter approach having the highest percentage of leakage (100%) (*p*-value = 0.046) (Tables 4, 5).

**Table 4:** Univariate analysis of the patients and clinical characteristics against postprocedural aortic status (presence of leakage/regurgitation) (*N* = 17).

Variables		Postprocedural aortic status				*p*-value	Phi/crammer's *V* values
		Without leakage/regurgitation		With leakage/regurgitation			
		Frequency	Row percentage	Frequency	Row percentage		
Age (Years)		76.4 (Mean)	7.1 (SD)^a^	80 (Mean)	8 (SD)^a^	0.442	^c^
Gender	Male	8	72.7%	3	27.3%	0.794	0.064
	Female	4	66.7%	2	33.3%		
Location	Japan	2	40.0%	3	60.0%	0.149	0.646
	Mexico	2	100.0%	0	0.0%		
	South Korea	1	100.0%	0	0.0%		
	China	6	85.7%	1	14.3%		
	France	1	100.0%	0	0.0%		
	Australia	0	0.0%	1	100.0%		
Functional status of aortic valve	Aortic regurgitation	4	100.0%	0	0.0%	0.066	0.477
	Aortic Stenosis	2	100.0%	0	0.0%		
	Both aortic stenosis and regurgitation	6	54.5%	5	45.5%		
Clinical presentation	Symptoms of heart failure	6	85.7%	1	14.3%	0.461	0.366
	Progressive dyspnea	3	60.0%	2	40.0%		
	Syncope	1	100.0%	0	0.0%		
	Symptomatic aortic stenosis and regurgitation	2	50.0%	2	50.0%		
Presence of hypertension as co-morbidity	Yes	5	83.3%	1	16.7%	0.380	0.207
	No	7	63.6%	4	36.4%		
Status of Left ventricular ejection fracture	Normal	5	83.3%	1	16.7%	0.295	−0.272
	Abnormal	4	100.0%	0	0.0%		
New York Heart Association (NYHA) Functional Classification	class III	6	85.7%	1	14.3%	0.383	0.218
	class IV	3	100.0%	0	0.0%		
Presence of calcification	Yes	5	71.4%	2	28.6%	0.118	−0.378
	No	5	100.0%	0	0.0%		
Annulus perimeter (mm)		81.6 (Mean)	10.4 (SD)^a^	81.2 (Mean)	3.0 (SD)^a^	0.667	^c^
Annulus area (mm^2^)		502.7 (Mean)	139.0 (SD)^a^	505.7 (Mean)	31.1 (SD)^a^	0.889	^c^
RCA height (mm)		11.7 (Mean)	2.7 (SD)^a^	11.8 (Mean)	^b^	1.000	^c^
LCA height (mm)		13.8 (Mean)	3.6 (SD)^a^	16.1 (Mean)	^b^	0.800	^c^

a SD = Standard deviation.

b Can't be calculated.

c Not applicable.

**Table 5:** Univariate analysis of the surgical characteristics against postprocedural aortic status x (presence of leakage/regurgitation) (*N* = 17).

Variables		Postprocedural aortic status				*p*-value	Phi/Crammer's *V* values
		Without leakage/regurgitation		With leakage/regurgitation			
		Frequency	Row percentage	Frequency	Row percentage		
QVA type (Hurwitz and Roberts classification)	Not identified	3	60.0%	2	40.0%	0.211	0.566
	A	4	100.0%	0	0.0%		
	B	2	40.0%	3	60.0%		
	C	1	100.0%	0	0.0%		
	D	1	100.0%	0	0.0%		
	F	1	100.0%	0	0.0%		
Surgery approach	Transfemoral	7	63.6%	4	36.4%	0.046*	0.528
	Transcatheter (TAVR)	0	0.0%	1	100.0%		
	Transapical	5	100.0%	0	0.0%		
Transcatheter aortic valve replacement (TAVR) devices used	J -valve	5	100.0%	0	0.0%	0.092	0.673
	Venus A-valve	1	50.0%	1	50.0%		
	Edwards SAPIEN 3	5	71.4%	2	28.6%		
	PORTICO transcatheter heart valve	0	0.0%	1	100.0%		
	Evolut R valve	0	0.0%	1	100.0%		
	Edwards SAPIEN XT valve	1	100.0%	0	0.0%		
Pre dilatation performed	Yes	4	57.1%	3	42.9%	0.064	−0.239
	No	4	80.0%	1	20.0%		

* *p*-value < 0.05.

### Risk of bias in included studies

Following the JBI Critical Appraisal Tools, we conducted a detailed risk of bias assessment separately for case reports and case series. The risk of bias in the included case reports is summarized in Table 6, and the risk of bias in the included case series is summarized in Table 7. The assessment reveals a generally low risk of bias across both the case reports and the case series included in this review. Most case reports provided comprehensive details on patient demographics, clinical history, diagnostic testing, and follow-up outcomes. Adverse events occasionally need to be explicitly detailed, which introduces some uncertainty. However, clear documentation of intervention methods and case-specific outcomes strengthens the credibility of these reports.

**Table 6:** The risk of bias of the included case reports.

Study ID	Demographics described	History timeline	Clinical condition	Diagnostic tests	Intervention description	Post-intervention condition	Adverse events	Takeaway lessons	Final
Bruno, 2024 (15)	Yes	Yes	Yes	Yes	Yes	Yes	No	Yes	Good
Yu Han, 2021 (19)	Yes	Yes	Yes	Yes	Yes	Yes	Unclear	Yes	Good
Tomoki Fukui, 2020 (10)	Yes	Yes	Yes	Yes	Yes	Yes	No	Yes	Good
Samuel Sidharta, 2015 (12)	Yes	Yes	Yes	Yes	Yes	Yes	No	Yes	Good
Chaodi Luo, 2021 (11)	Yes	Yes	Yes	Yes	Yes	Yes	Unclear	Yes	Good
Rie Aoyama, 2018 (13)	Yes	Yes	Yes	Yes	Yes	Yes	Yes	Yes	Good
Cheol Woong Yu, 2014 (14)	Yes	Yes	Yes	Yes	Yes	Yes	No	Yes	Good
Henri Benkemoun, 2020 (16)	Yes	Yes	Yes	Yes	Yes	Yes	No	Yes	Good
Masao Takahashi, 2022 (17)	Yes	Yes	Yes	Yes	Yes	Yes	No	Yes	Good
Daisuke Sato, 2022 (18)	Yes	Yes	Yes	Yes	Yes	Yes	Yes	Yes	Good

**Table 7:** The risk of bias of the included case series.

Study ID	Inclusion criteria	Condition measured reliably	Valid methods	Consecutive inclusion	Complete inclusion	Demographic reporting	Clinical info	Outcome reporting	Site demographics	Appropriate stats	Final
Yang Liu, 2022 (9)	Yes	Yes	Yes	Unclear	Yes	Yes	Yes	Yes	Yes	Yes	Good

For the case series, clear inclusion criteria, valid measurement methods, and complete outcome reporting further support a low bias risk. Though consecutive inclusion was only sometimes specified, demographic and clinical details were well-reported, and appropriate statistical analyses were applied where needed. Overall, the studies are methodologically sound, minimizing concerns related to potential bias in reporting or study design.

## Discussion

This meta-analysis examined the outcomes of transcatheter aortic valve replacement (TAVR) in patients with quadricuspid aortic valves (QAV), focusing on procedural success, postoperative complications, and the impact of valve morphology on outcomes. The findings affirm that TAVR offers a feasible alternative to surgical aortic valve replacement (SAVR), particularly in high-risk patients. The inclusion of newer valve generations and procedural improvements has contributed to high procedural success rates and satisfactory hemodynamic outcomes.

The present analysis demonstrated a high procedural success rate of 100%, consistent with previous studies of Liu Y. et al. (9) and Bruno et al. (15) evaluating TAVR in both tricuspid and quadricuspid aortic valves. This could be explained due to the subjective decision by the surgeons themselves rather than by absence of complications. The 30-day mortality rate of 4.6% is comparable to that observed in other high-risk populations undergoing TAVR, confirming its viability for patients unsuitable for SAVR. No significant differences were noted in procedural success or mortality between valve types (e.g., CoreValve vs. SAPIEN), corroborating earlier findings that these valves are similarly effective (15). This reaffirms the notion that valve selection should be patient-specific, with morphology and anatomical characteristics guiding decision-making.

Furthermore, Moderate or greater paravalvular leak (PVL) occurred in 18.2% of patients, which is expected to be slightly higher in QAV patients due to their complex valve anatomy, as reported by Sidharta S. et al. (12). This rate is somewhat elevated compared to patients with tricuspid valves, likely reflecting the irregular shape of QAVs. Recent iterations of valves, such as SAPIEN 3 and CoreValve Evolut, have incorporated design features aimed at reducing PVL, such as sealing skirts (17).

Pacemaker implantation occurred in 22.3% of patients, consistent with prior TAVR studies. Although CoreValve recipients were slightly more prone to requiring a pacemaker, this difference was not statistically significant once baseline characteristics were adjusted (15). The radial force exerted by self-expanding valves may explain the slightly higher incidence of pacemaker implantation in CoreValve recipients (18).

Hemodynamic improvements were marked, with mean transvalvular gradients decreasing from 55 mmHg to 11 mmHg post-procedure. This reduction highlights the effectiveness of TAVR in improving valve function in Liu Y. et al. (9). Additionally, left ventricular ejection fraction (LVEF) improved in 39% of patients, although 13% did not experience improvement, likely due to pre-existing ventricular dysfunction or delayed intervention (13).

Moreover, no significant differences in procedural success or mortality were associated with valve type. However, patients with Type B QAV were more likely to experience moderate PVL compared to other morphologies, underscoring the importance of valve morphology in TAVR outcomes (10). This finding aligns with reports that patients with irregular valve shapes, such as QAV, are at higher risk of complications like PVL (11). Special consideration should be given to valve morphology, as certain types (e.g., Type B) may predispose to higher rates of paravalvular leak. Thus, tailoring valve selection and implantation techniques to specific anatomical challenges may reduce complications (10, 11).

Hospital stays ranged from 2 to 9 days, with shorter stays associated with fewer post-operative complications. Follow-up durations extended from 1 month to 4 years, revealing that improvements in hemodynamic performance and functional status were generally sustained in the mid-term (16). However, longer-term durability data beyond 4 years remain limited, and future research should aim to clarify valve longevity and its impact on patient survival (18).

Notably, in the present analysis, the surgical approach was significantly associated with post-procedural aortic valve status, with the transcatheter approach demonstrating the highest incidence of paravalvular leak (*p* = 0.046). This suggests that patients treated via non-standard or alternative transcatheter access may be at increased risk of suboptimal valve sealing. The higher leakage rate could reflect technical challenges associated with less direct access routes, suboptimal coaxial alignment, or anatomical limitations inherent to complex QAV morphology. Although the number of patients undergoing the transcatheter approach was small, these findings underscore the importance of careful procedural planning, particularly with respect to access strategy, in QAV patients (14, 16, 17).

This meta-analysis confirms that TAVR is a highly effective and relatively safe intervention for patients with aortic stenosis and regurgitation, including those with QAV. However, complications such as PVL warrant ongoing attention, particularly in patients with anatomically complex valves like QAV (13). As newer valve designs continue to evolve, further research into optimizing valve selection and procedural techniques will be crucial to reducing these complications.

This study has several important limitations. First, the small sample size (17 patients) limits the statistical power and generalizability of the findings. Second, inclusion was restricted to English-language publications, introducing potential language bias and possibly omitting relevant non-English studies. Third, reliance on case reports and small case series introduces selection and publication biases, as these studies are more likely to report favorable outcomes. Additionally, considerable heterogeneity in patient characteristics, procedural techniques, and reporting standards may have affected outcome consistency, particularly regarding paravalvular leak rates. Missing data in several clinical and procedural variables further limited the scope of statistical analysis. Moreover, the available follow-up periods were relatively short in most cases, preventing firm conclusions about the long-term durability of transcatheter valves in QAV patients. Although a random-effects model was used to account for heterogeneity, inherent variability across case-based studies remains a source of potential bias. Future larger prospective studies and multicenter registries are needed to validate these preliminary findings and optimize management strategies for this unique patient population.

## Conclusion

TAVR remains a viable and increasingly popular treatment for high-risk patients with aortic valve disease, including those with QAV. It demonstrates high procedural success but a notable risk of paravalvular leak. However, our findings were based on a small number of cases. Larger studies with longer follow-up are needed to optimize procedural strategies.

## Data Availability

The original contributions presented in the study are included in the article/Supplementary Material, further inquiries can be directed to the corresponding author.
