# Correction: Transcatheter aortic valve replacement in a quadricuspid aortic valve: a systematic review of case reports

**DOI:** 10.3389/fcvm.2026.1816950

**Published:** 2026-04-02

**Authors:** Mostafa A. Khalifa, Hashim Talib Hashim, Aya Ahmed Shimal, Fathima Raahima Riyas Mohamed, Srinithi Ragunathan, Ahmed Sermed Al Sakini, Mohamed H. Elbadawi, Mohammed Rushdhi Irfan, Ibrahim Moqbel, Marwah Mohammed Almualed, Mohammedbaqer Al-Ghuraibawi, Batool S. Al-Aboudi

**Affiliations:** 1 Faculty of Medicine, Cairo University, Cairo, Egypt; 2 College of Medicine, University of Warith Al-Anbiyaa, Karbala, Iraq; 3 College of Medicine, University of Baghdad, Baghdad, Iraq; 4 College of Medicine, Alfaisal University, Riyadh, Saudi Arabia; 5 Panimalar Medical College and Research Institute, Chennai, India; 6 Faculty of Medicine, University of Khartoum, Sudan; 7 College of Medicine, King Abdulaziz University, Jeddah, Saudi Arabia; 8 Faculty of Medicine, University of Kufa, Najaf, Iraq

**Keywords:** aortic stenosis, procedural success, quadricuspid aortic valves, QVA, transcatheter aortic valve replacement

The title of this article was erroneously given as: Transcatheter aortic valve replacement in a quadricuspid aortic valve: a systematic review and meta-analysis. The correct title of the article is Transcatheter aortic valve replacement in a quadricuspid aortic valve: a systematic review of case reports.

In the abstract, “A random-effects model was used for meta-analysis” has been corrected to read:

“A descriptive pooled analysis of individual participant data using a one-stage approach was performed, along with appropriate univariate tests”

The original version of this article has been updated.

